# A new indocyanine green fluorescence lymphography protocol for identification of the lymphatic drainage pathway for patients with breast cancer-related lymphoedema

**DOI:** 10.1186/s12885-019-6192-1

**Published:** 2019-10-22

**Authors:** Hiroo Suami, Asha Heydon-White, Helen Mackie, Sharon Czerniec, Louise Koelmeyer, John Boyages

**Affiliations:** 1 Australian Lymphoedema Education Research Treatment (ALERT) Program, Faculty of Medicine and Health Sciences, Macquarie University, Level 1, 75 Talavera Rd, Sydney, NSW 2109 Australia; 2 Mt Wilga Private Hospital, Hornsby, NSW Australia

**Keywords:** Lymphoedema, Lymphography, Lymphatic system, Manual lymphatic drainage, Breast cancer, Molecular imaging

## Abstract

**Background:**

Breast cancer related lymphoedema (BCRL) is a common side effect of cancer treatment. Recently indocyanine green (ICG) fluorescent lymphography has become a popular method for imaging the lymphatics, however there are no standard protocols nor imaging criteria. We have developed a prospective protocol to aid in the diagnosis and therapeutic management of BCRL.

**Methods:**

Lymphatic imaging procedures were conducted in three phases. Following initial observation of spontaneous movement of ICG in phase one, manual lymphatic drainage (MLD) massage was applied to facilitate ICG transit via the lymphatics in phase two. All imaging data was collected in phase three. Continuous lymphatic imaging of the upper limb was conducted for approximately an hour and lymphatic drainage pathways were determined. Correlations between the drainage pathway and MD Anderson Cancer Centre (MDACC) ICG lymphoedema stage were investigated.

**Results:**

One hundred and three upper limbs with BCRL were assessed with this new protocol. Despite most of the patients having undergone axillary node dissection, the ipsilateral axilla drainage pathway was the most common (67% of upper limbs). We found drainage to the ipsilateral axilla decreased as MDACC stage increased. Our results suggest that the axillary pathway remained patent for over two-thirds of patients, rather than completely obstructed as conventionally thought to be the case for BCRL.

**Conclusions:**

We developed a new ICG lymphography protocol for diagnosing BCRL focusing on identification of an individual patient’s lymphatic drainage pathway after lymph node surgery. The new ICG lymphography protocol will allow a personalised approach to manual lymphatic drainage massage and potentially surgery.

## Background

Breast-cancer related lymphoedema (BCRL) is a common side effect of cancer treatment causing physical, functional, psychological and financial challenges for individuals and impacting their quality of life [1–4]. Lymphoscintigraphy is the standard technique in lymphatic imaging for diagnosing lymphoedema [5, 6]. Although no universal protocol exists, three imaging criteria are used in diagnosis; delayed transit of radioactive tracer compared to the unaffected limb and presence of dermal backflow, the accumulation of the tracer in the dermal lymphatics and absence or reduced number of draining lymph nodes.

Recently, Indocyanine Green (ICG) lymphography has become an alternate popular method for imaging the lymphatics. ICG lymphography was initially used for breast sentinel node biopsy [7]. Its application then extended to lymphoedema diagnosis and mapping lymphatic vessels prior to lymphovenous anastomosis (LVA) surgery [8–10].

The above lymphoscintigraphy criteria for lymphoedema diagnosis cannot be applicable for ICG lymphography because penetration of the near infrared rays is limited to 2 cm from the skin surface making it difficult and inconsistent to identify lymph nodes [11]. However, ICG lymphography has some advantages for lymphatic imaging over lymphoscintigraphy. ICG is a water-based solution and therefore travels faster via the lymphatics compared to technetium 99 m-sulfer colloid commonly used as the radionuclide for lymphoscintigraphy, enabling high resolution, and real time imaging. Furthermore, ICG is not radioactive and does not require special storage precautions [12].

Due to the requirement of new imaging criteria for the diagnosis of lymphoedema using ICG lymphography, we have developed a prospective protocol to aid in the diagnosis of BCRL, assist decision making for therapeutic management including ICG-directed manual lymphatic drainage (MLD) massage and define selection criteria for surgical options. The aim of this study to summarise initial findings obtained by the new ICG lymphography protocol in breast cancer related lymphoedema.

## Methods

A retrospective cohort audit was conducted, reviewing prospectively collected data from patients with BCRL who underwent ICG lymphography at the Australian Lymphoedema Education, Research and Treatment (ALERT) clinic at Macquarie University (MQ) between February 2017 and April 2018. Data were sourced from electronic medical records and this audit was approved by MQ Health Ethics Committee (Reference: MQCIA2018017). Written informed consent was obtained from all patients in this study.

In three patients for the pilot study, we repeated the ICG imaging after 24 h and compared with the images obtained with this protocol. If the patients had previous lymphoscintigraphy in the affected limb, both lymphoscintigraphy and ICG lymphography images were compared.

### ALERT ICG lymphography imaging protocol

**Fig. 1:**
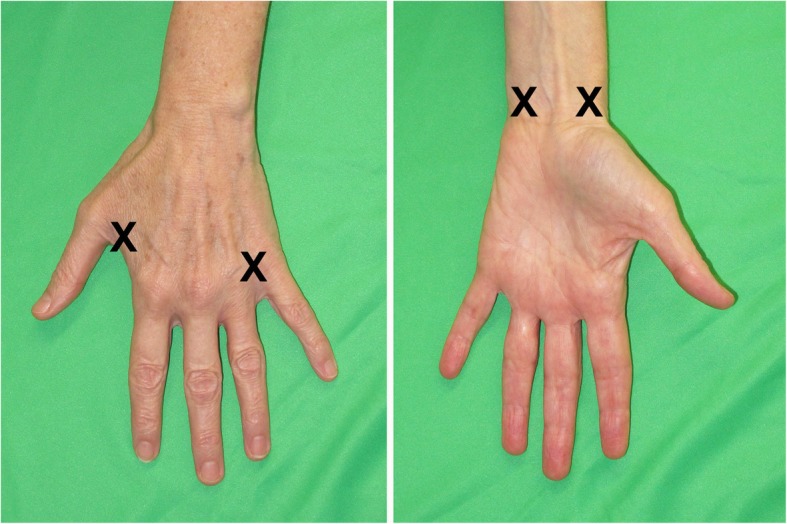
ICG injection sites

The near infrared camera system (PDE Neo II; Hamamatsu Photonics K.K.) was used for this study. Indocyanine Green (Verdye 25 mg; Diagnostic Green GmbH) was mixed with 5 ml of saline. Four injection sites were used in the distal aspect of the upper limb on the affected side: first and fourth web spaces and ulnar and radial volar wrist regions (Fig. 1). These circumferential injection sites were chosen based on our previous cadaveric lymphatic anatomy studies which demonstrated that lymphatic vessels originate individually and have few interconnections [13–15]. ICG lymphography was only applied for the affected limb instead of imaging the unaffected limb as a control because our cadaveric studies confirmed uniformity of the lymphatic drainage pathways in normal anatomy between individuals [13–15]. Further we considered bilateral imaging to be more costly, time-consuming and more stressful for the patient.

Intradermal injections were performed with a 30-gauge needle and a 1 ml syringe. At each injection site 0.05-0.1 ml (0.25-0.5 mg) of ICG solution was administered. A cryogenic numbing device (CoolSense; CoolSense Medical Ltd.) was used immediately before each injection to reduce needle discomfort [16].

Lymphatic scanning using the near infrared camera commenced immediately after the injections and imaging data was recorded using a digital video recorder (MDR-600HD: Ikegami Tsushinki Co., Ltd.). Lymphatic imaging of the upper limb was continuously conducted in each upper limb for approximately an hour.

### Imaging procedures

Lymphatic scanning was conducted in three phases.

#### Phase one

Observation of any spontaneous movement of ICG via the lymphatics for approximately 10 min. Patients were encouraged to clench and unclench their hand ten times to facilitate lymphatic uptake of the ICG.

#### Phase two

Manual lymphatic drainage (MLD) massage was then performed by an accredited lymphoedema therapist to facilitate ICG transit via the lymphatics. This MLD is undertaken by the therapist and the patient’s real time visualisation of the lymphatic vessels and areas of dermal backflow provides patient feedback of direction, speed and skin pressure required to move the ICG dye. Scanning focused on identifying lymphatic vessels and the competency of their valves, direction of dermal backflow extension, and identifying lymph nodes. We found that MLD facilitated dye movement more efficiently compared to post-injection exercise and delayed scanning although this was not formally evaluated. When lymphatic vessels were identified, their course was marked on the patient’s skin with a coloured pen (Fig. 1a). Phase two continued until the dissemination of ICG reached a plateau without any further movement, usually between 30 and 45 min.

#### Phase three

**Fig. 2:**
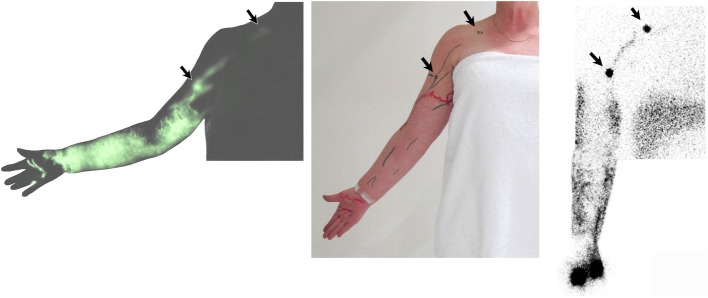
Comparison of ICG lymphography and tracing photo (left and centre) and Lymphoscintigraphy image (right) in the same patient

Demarcation lines of dermal backflow were marked on the skin, and collection of imaging data through still photography with both near infrared and digital cameras were taken. (Fig. 2 left and centre, and 3). Phase three takes approximately 15 min.

### Imaging data analysis

Still ICG images were montaged with image editing software (Photoshop CC; Adobe Systems) to provide an image of the whole upper limb. The lymphatics were designated into two categories; lymphatic vessels in the subcutaneous tissue and dermal backflow which is reflux of lymph fluid into dermal lymphatics. Lymphoedema was diagnosed by the presence of dermal backflow. Although ICG lymphography was considered mainly for imaging the superficial lymphatics, the following observation helped us to interpret the images. For example, if the epitrochlear lymph node was identified in the medial elbow, the efferent lymphatic vessel of the node was known to run along the brachial artery in the upper arm [17]. Although the ICG near infrared signal in the upper arm was often missing, we could identify the signal in the operated axilla because of reduced soft tissue. Thus we could confirm that the lymphatic drainage pathway drained to the ipsilateral axilla.

Lymphatic drainage pathways were determined by the location of identified lymph nodes or extension of ICG to the skin regions via dermal backflow or lymphatic vessels where lymph nodes were located underneath. Lymphoedematous upper limbs were also classified by MDACC stage as 0: normal lymphatics, Stage 1: many patent lymphatic vessels with minimal patchy dermal backflow, Stage 2: moderate number of patent lymphatic vessels with segmental dermal backflow, Stage 3: few patent lymphatic vessels with extensive dermal backflow involving the entire arm, Stage 4: no patent lymphatic vessels seen with dermal backflow involving the entire arm with extension to the dorsum of the hand and Stage 5: ICG does not move from injection sites [18, 19].

## Results

One hundred and seven upper limbs at-risk or affected by BCRL were examined in 103 patients (unilateral: 99, bilateral: 4). Three patients who were previously diagnosed with unilateral BCRL were found by our imaging criteria to demonstrate normal lymphatics (MDACC Stage 0) and an additional bilateral patient who had normal lymphatics on the side of the sentinel node biopsy were excluded. Our study cohort therefore consisted of 103 upper limbs (unilateral 97, bilateral 3) examined in 100 patients. Patient characteristics are described in Table 1. Of note, axillary dissection was performed in 99 limbs, 2 had a sentinel node biopsy and for 2 patients the extent of axillary surgery was unknown.

**Table 1:** Patient characteristics

Characteristic	
Age (yr), mean (SD)	57.73 (±9.78)
Time since cancer diagnosis (yr) mean (SD)	7.26 (±6.88)
Breast Surgery type *n* (%)	
Mastectomy	76 (73.78)
Lumpectomy/WLE	25 (24.27)
NSM and implant	2 (1.94)
Axilla surgery type *n* (%)	
ALND	99 (96.11)
SNB	2 (1.94)
Unknown	2 (1.94)
Adjuvant therapy *n* (%)	
Radiotherapy	84 (81.55)
Taxane chemotherapy	62 (60.19)

*WLE* Wide Local Excision, *NSM* Nipple Sparing Mastectomy, *ALND* Axillary Lymph Node Dissection, *SNB* Sentinel Node Biopsy, *yr* year, *n* number

### ICG lymphography findings

We could frequently specify sites in the upper limb where the lymphatic vessel was obstructed. Dermal backflow was identified at these obstruction sites extending through the dermal lymphatics (Additional file 1: Video S1).

ICG lymphography demonstrated that MLD could facilitate transit of ICG via dermal backflow and lymphatic vessels (Additional file 2: Video S2 and Additional file 3: Video S3). ICG moved slower in dermal backflow and faster in the lymphatic vessel probably due to the calibre and contractility of the lymphatic vessels. When MLD was performed to areas of dermal backflow, ICG moved directionally instead of extending in all directions. We recognised two patterns of the directional ICG spread in dermal backflow. First, dermal backflow was observed initially at a site of lymphatic vessel obstruction and moved towards an adjacent patent lymphatic vessel. Dermal backflow worked as a detour route acting as a bridge between the obstructed and patent lymphatic vessels. Second, when no patent vessels remained in the limb, dermal backflow extended directly to a lymph node region such as the ipsilateral axilla, clavicular or parasternal regions.

We also defined the demarcation line of dermal backflow at the end of the procedure (Fig. 1a in red). Of the three patients who underwent both ICG lymphography and lymphoscintigraphy there was good consistency of the presence of dermal backflow, identification of lymph nodes and lymphatic drainage pathways between the two techniques. However, real-time ICG lymphography allowed precise demarcation of dermal backflow.

In three patients who repeated the ICG imaging after 24 h, we found that, although the delayed-images had faded slightly, the demarcation lines of the dermal backflow and the lymphatic drainage pathways were identical. This suggests that our protocol of an approximately one hour ICG imaging session combined with MLD is sufficient to gain maximum information for patients with BCRL.

**Fig. 3:**
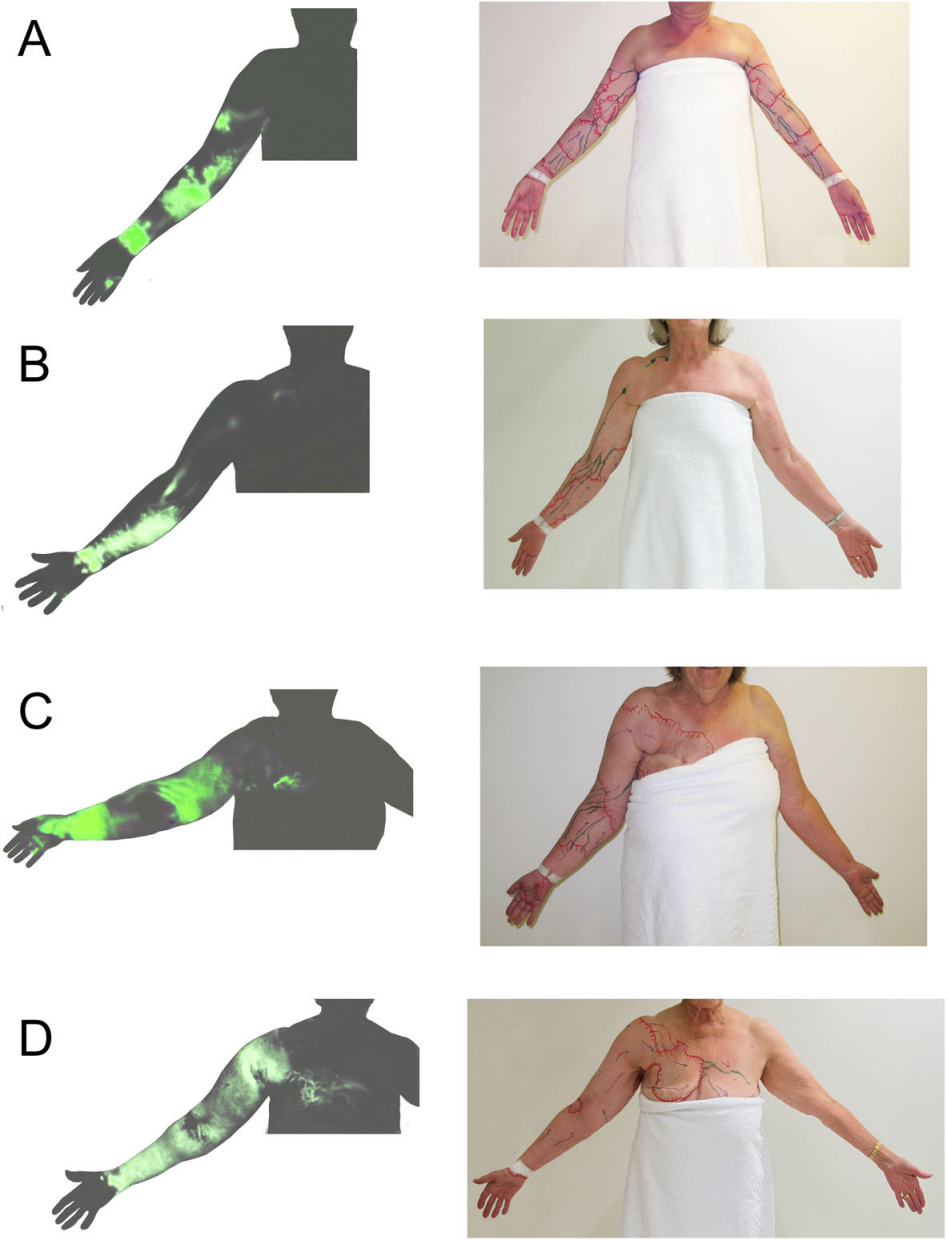
Patterns of drainage pathway in ICG lymphography images (left) and tracing photos (right): **a** ipsilateral axilla, **b** clavicular, **c** parasternal, and **d** contralateral axilla

Of the103 upper limb examined patients, none were classified into MDACC Stage 5. 34 upper limbs (32%) demonstrated more than one drainage pathway. Variations of drainage pathway patterns are summarised in Table 2 and Fig. 3. Overall the percentage of drainage to the ipsilateral axilla was 67%. We found drainage to the ipsilateral axilla decreased as MDACC stage increased. As drainage to the axilla decreased by stage, we found drainage to the ipsilateral clavicular pathway increased reaching a peak of 55% for patients with MDACC Stage 3 lymphoedema. Patients with MDACC Stage 4 lymphoedema had the highest rate of drainage to the parasternal pathway and contralateral axilla (17%). In these cases, dermal backflow in the upper limb extended to the anterior midline of the chest and rerouted to the contralateral axilla via the intact lymphatic vessels in the contralateral breast. Of note, if there was a functional pathway to the proximal region of the ipsilateral upper limb, ICG did not extend beyond this region. For example, dermal backflow extended either to the parasternal region or to the contralateral axilla only when the pathway to the ipsilateral axilla or clavicular region was obstructed.

**Table 2:** Lymphatic Drainage Pathways

ICG drainage pathways							
MDACC stage	No.	Ipsilateral axilla	Clavicular	Parasternal	Contralateral axilla	Ipsilateral Inguinal	Unknown
1	19	95%	21%	5%	0%	0%	0%
2	46	61%	52%	7%	2%	0%	0%
3	20	70%	55%	5%	5%	0%	0%
4	18	50%	17%	17%	17%	0%	11%
Total	103	67%	41%	8%	5%	0%	11%

## Discussion

The diagnosis of lymphoedema is often difficult by physical examination alone, especially in early stages. Patients with BCRL often complain, for example, of discomfort in specific areas of their upper limb instead of uniform changes or swelling in the whole limb. In this study, we introduced a new ICG lymphography protocol for the upper limb to help to identify areas with underlying anatomical changes that occur in lymphoedema. Our previous review study found that ICG lymphography had the potential benefit to elucidate the relationship between lymphatic drainage pathway and severity of lymphoedema [20]. The drainage pathway to the clavicular region was commonly identified for patients with MDACC ICG Stage 2 or 3 lymphoedema and occurred in 52-55% of patients respectively. It was apparent that sternal and contralateral pathway groups were found in Stage 4 lymphoedema (Table 2).

Lymphoscintigraphy has been the standard imaging examination for lymphoedema [21, 22]. However, conventional lymphoscintigraphy protocols for lymphoedema do not include identification of the lymphatic drainage pathways because spontaneous transit of viscous radionuclide tracer cannot reach lymph nodes in lymphoedematous limbs constantly. In recent, stress-lymphoscintigraphy including exercise was introduced to improve lymphatic visualization but radiation exposure prevents applying MLD for facilitating tracer transit [23, 24]. In comparison ICG mixture moves faster than the lymphoscintigraphy tracer and facilitation of ICG transit with MLD can reduce examination time to specify the lymphatic drainage pathway and provides additional direct therapeutic guidance to the patient and the therapist.

Another advantage of ICG lymphography is that some patients may indeed not suffer from lymphoedema. In our study four limbs in four patients were diagnosed as not having BCRL as they had normal lymphatic drainage without any dermal backflow. Future research should address the correlation of ICG lymphography with subclinical lymphedema detected by bioimpedance spectroscopy. Further, there is often a misconception that lymphatic drainage occurs away from the dissected axilla. In Abe’s lymphangiography studies 13 of 19 patients (68%) showed patent lymph vessels passing through the axilla [25]. This was almost identical to our rate of 67% indicating that the ipsilateral axilla is still considered as a vital pathway.

Conventionally, BCRL was thought to be caused by the complete obstruction of the lymphatic drainage to the ipsilateral axilla secondary to surgical and/or radiation intervention. Our results contradict this notion and suggest that the axillary pathway was restricted functionally instead of complete obstruction in over two-thirds of patients.

## Conclusion

We developed a new ICG lymphography protocol for diagnosing BCRL focusing on identification of an individual patient’s lymphatic drainage pathway after lymph node surgery to guide MLD and to assist with selection criteria for lymphatic microsurgery. ICG imaging combined with MLD will allow a personalised approach to lymphoedema care.

## Supplementary information

**Additional file 1.** Dermal backflow was identified with ICG lymphography.

**Additional file 2.** Gentle MLD could move ICG via lymphatic vessels in mild lymphoedema.

**Additional file 3.** Firmer MLD could move ICG via dermal backflow in severe lymphoedema.

## Data Availability

The datasets used and analysed during the current study are available from the corresponding author on reasonable request.

## Abbreviations

ALERT: Australian lymphoedema education, research, and treatment
BCRL: Breast cancer related lymphoedema
ICG: Indocyanine green
MDACC: MD Anderson Cancer Centre
MLD: Manual lymphatic drainage
